# Metabolomic alterations associated with Behçet’s disease

**DOI:** 10.1186/s13075-018-1712-y

**Published:** 2018-09-24

**Authors:** Wenjie Zheng, Xiuhua Wu, Maryam Goudarzi, Jing Shi, Wei Song, Chaoran Li, Jinjing Liu, Hua Chen, Xuan Zhang, Xiaofeng Zeng, Heng-Hong Li

**Affiliations:** 1 Department of Rheumatology and Clinical Immunology, Key Laboratory of Rheumatology and Clinical Immunology, Peking Union Medical College Hospital, Peking Union Medical College and Chinese Academy of Medical Sciences, Ministry of Education, Beijing, China; 2 Department of Rheumatology, General Hospital of Tianjin Medical University, Tianjin, China; 3 Georgetown University Medical Center, Georgetown University, Washington, DC USA; 4 Central Research Laboratory, Peking Union Medical College Hospital, Peking Union Medical College and Chinese Academy of Medical Sciences, Ministry of Education, Beijing, China

**Keywords:** Behçet’s disease, Autoinflammatory disease, Biomarker, Metabolomics, Lipidomics

## Abstract

**Background:**

The diagnosis of Behçet’s disease (BD) remains challenging due to the lack of diagnostic biomarkers. This study aims to identify potential serum metabolites associated with BD and its disease activity.

**Methods:**

Medical records and serum samples of 24 pretreated BD patients, 12 post-treated BD patients, and age-matched healthy controls (HC) were collected for metabolomics and lipidomics profiling using UPLC-QTOF-MS and UPLC-QTOF-MS^E^ approaches. Additionally, serum samples from an independent cohort of BD patients, disease controls including rheumatoid arthritis (RA), systemic lupus erythematosus (SLE), Takayasu’s arteritis (TA), Crohn’s disease (CD) patients, and HC were collected for further validation of two potential biomarkers using UPLC-QTOFMS analysis.

**Results:**

Unsupervised principal component analysis (PCA) showed a clear separation of metabolomics profiles of BD patients from HC. Statistical analysis of the data revealed differential metabolites between BD patients and HC. The serum levels of some phosphatidylcholines (PCs) were found to be significantly lower in BD patients, while the levels of several polyunsaturated fatty acids (PUFAs) were increased markedly in the BD group compared with HC. Furthermore, the serum level of two omega-6 PUFAs, linoleic acid (LA) and arachidonic acid (AA), were dramatically decreased in patients with remission. A validation cohort confirmed that the serum LA and AA levels in BD patients were significantly higher than those in HC and patients with RA, SLE, TA, and CD. In addition, receiver operating characteristic (ROC) analysis indicated good sensitivity and specificity.

**Conclusions:**

The serum metabolomics profiles in BD patients are altered. Serum LA and AA are promising diagnostic biomarkers for BD.

**Electronic supplementary material:**

The online version of this article (10.1186/s13075-018-1712-y) contains supplementary material, which is available to authorized users.

## Background

Behçet’s disease (BD) is a chronic multisystem inflammatory disorder characterized by recurrent oral and genital ulceration, uveitis, and skin lesions. Currently, the diagnosis of BD is primarily based on clinical manifestations, and no diagnostic biomarkers are available. BD is a multisystem vasculitis, so both arteries and veins of all sizes may be involved [1]. Since the clinical symptoms of BD are variable, it is sometimes difficult to distinguish it from other diseases such as inflammatory bowel disease and Reiter’s syndrome. As a result, early diagnosis remains a challenge in clinical practice.

Metabolomics, an emerging “omics” science, uses state-of-the-art quantitative analysis approaches and advanced bioinformatic methods to characterize the metabolome. It reflects both physiological and pathological states, and it may detect the alterations of affected metabolites at the early stages of disease due to its great sensitivity [2]. Metabolomic methods have been used for evaluating clinical diagnosis and therapeutic treatment in a variety of diseases, such as cancer, diabetes, multiple sclerosis, primary biliary cirrhosis, and autoimmune hepatitis [3–7]. Metabolic abnormalities in BD remain elusive. Given that serum is an accessible and informative biofluid, this study aims to identify serum metabolites in BD and to elucidate the metabolites responsive to treatment using a metabolomics approach.

## Methods

### Patients and controls

For metabolomics and lipidomics profiling, 24 BD patients and 25 gender- and age-matched healthy controls (HC) (without a personal or family history of autoimmune diseases) were enrolled from Peking Union Medical College Hospital (PUMCH) between March 2014 and November 2014. For further validation, an independent cohort of BD (*n* = 25), rheumatoid arthritis (RA) (*n* = 12), systemic lupus erythematosus (SLE) (*n* = 12), Takayasu’s arteritis (TA) (*n* = 15), and Crohn’s disease (CD) (n = 15) patients, and 19 HC were enrolled from March 2014 to July 2018. All BD patients fulfilled the 1990 International Study Group BD criteria or the new International Criteria for Behçet’s Disease (ICBD) [8, 9]. RA, SLE, TA and CD patients fulfilled their respective diagnostic and classification criteria [10–13]. All participants underwent a clinical evaluation, and hospital records were reviewed. The following data were collected: disease duration, clinical manifestations, erythrocyte sedimentation rate (ESR)/C-reactive protein (CRP) level, and treatment. BD disease activities were evaluated according to the BD Current Activity Form 2006 (BDCAF 2006; http://www.behcetdiseasesociety.org/behcetwsData/Uploads/files/BehcetsDiseaseActivityForm.pdf).

This study was carried out in accordance with the recommendations of the institutional committee for the Protection of Human Subjects from PUMCH. All subjects gave written informed consent in accordance with the Declaration of Helsinki. The protocol was approved by the institutional committee for the Protection of Human Subjects from PUMCH. All methods were performed in accordance with the relevant guidelines and regulations.

### Sample preparation for metabolomics/lipidomics profiling

Sterile siliconized 0.6-mL Eppendorf tubes were used for sample preparation, and 25 μL serum was added to the tubes followed by 100 μL cold chloroform/methanol (2/1) containing lipid standards at predetermined concentrations as described previously [14]. The mixture was vortexed for 30 s at room temperature and then centrifuged at 13,000×g for 5 min to separate the polar and nonpolar species. Upper and lower phases were collected separately and transferred to new tubes. The white interphase was discarded. The collected samples were dried using a speed vacuum. The pellet of the upper phase, which primarily contained polar metabolites, was resuspended in 200 μL 50% methanol for metabolomic profiling. The lower phase was resuspended in 200 μL isopropanol/acetonitrile/water (50/25/25) for lipidomic analysis.

### UPLC-QTOF-MS and UPLC-QTOF-MS^E^ analysis

The chromatographic and mass spectrometric parameters were used as described previously [15]. The ultra-performance liquid chromatography (UPLC) column eluent was introduced directly into the mass spectrometer by electrospray. For metabolomic profiling, a 2-μL sample was injected onto a reverse-phase ACQUITY BEH C_18_ 50 × 2.1 mm 1.7-μm column (Waters Corp., Milford, MA) using an ACQUITY UPLC system (Waters Corp., Milford, MA). For lipidomics profiling, an Acquity CSH C_18_ 50 × 2.1 mm 1.7-μm column (Waters Corp., Milford, MA) was used in the UPLC-quadrupole time-of-flight mass spectrometry (UPLC-QTOF-MS^E^) analysis. MS^E^ is a technique by which both the precursor and fragment mass spectra are acquired by alternating between high and low collision energy during a single chromatographic run. Mass spectrometric analysis was performed on a XEVO G2 QTOF (Waters) operating in both positive and negative modes. Accurate mass was maintained by introducing the LockSpray interface of sulfadimethoxine (311.0814 [M + H]+ or 309.0658 [M − H]−) at a concentration of 250 pg/μL in 50% aqueous ACN at a rate of 150 μL/min. For biomarker validation, a 2-μL sample was injected into a reverse-phase ACQUITY HSS T3 C18 100 × 2.1 mm 1.7-μm column (Waters Corp., Milford, MA) and analyzed using the consistent UPLC-QTOF-MS system. The mobile phase consisted of acetonitrile (A) and water containing 0.1% (v/v) formic acid (B), while the gradient elution program (0.0–18.0 min, 10%–95% A; 18.1–20.0 min, 100% A) was applied for favorable separation. The flow rate was set at 0.4 mL/min. The column temperature was 40 °C. All chromatograms and mass spectrometric data were acquired in centroid mode using the MassLynx software (Waters Corp., Milford, MA).

### Data processing and multivariate data analysis

Raw mass spectrometric data were processed using Progenesis QI software (Nonlinear Dynamics, Durham, NC) to generate a data matrix that consisted of the retention time, m/z value, and the normalized peak area. Statistical analysis and putative ion identification on the postprocessed data were conducted using MetaboLyzer [16]. Statistically significant ions were putatively identified in MetaboLyzer, which utilizes the Human Metabolome Database (HMDB), LipidMaps, and the Kyoto Encyclopaedia of Genes and Genomes (KEGG) database [8] while accounting for possible adducts, H^+^, Na^+^, and NH4^+^ in the ESI^+^ mode, and H^–^ and Cl^–^ in the ESI^–^ mode. The m/z values were compared with the exact mass of small molecules in the databases, from which putative metabolites were identified with a mass error of 10 ppm or less. KEGG annotated pathways associated with these putative metabolites were also identified. Lipid ions were validated with the fragmentation in MS^E^ results based on their identifier fragments and retention time with the help of nonendogenous lipid standards from each lipid class and/or the comparison of tandem mass spectrometry (MS/MS) fragments with reference spectra provided in METLIN and LipidMaps databases. SIMCA-P+ (Umetrics, Umea, Sweden) was used for principal component analysis (PCA). The heat map displaying the relative levels of differential metabolites was generated by the Random Forests (RF) algorithm as explained in detail in previous studies [17]. MS data acquired in negative ion mode were employed for quantitation of arachidonic acid (AA) and linoleic acid (LA).

### Sample preparation for biomarker verification

For the preparation of calibration samples, reference substances of rosmarinic acid, AA, and LA were purchased from Sigma-Aldrich Company (MO, USA). Rosmarinic acid was dissolved in methanol to produce the internal standard (IS) solution at the concentration of 1 μg/mL. The reference solutions of the two targeted metabolites, AA and LA, at the stock concentrations of 1/1000 (v/v) for each were serially diluted with the IS solution to produce a series of calibration standard solutions. All calibration standard solutions were sealed and stored at 4 °C until use.

For serum samples, 100 μL serum was added to the tube, followed by 400 μL cold methanol. The mixture was vortexed for 30 s at room temperature and then centrifuged at 13,000×g at 4 °C for 5 min to precipitate the protein. The supernatant was transferred to the Eppendorf tube, dried using a speed vacuum at 25 °C, and then dissolved in 100 μL of the IS solution for LC/MS analysis.

### Statistical analysis

Experimental values are presented as mean ± SD. Statistical analysis was performed using GraphPad Prism (San Diego, CA). The significance of the metabolite level was determined using a two-tailed student *t* test. *P* values less than 0.05 were considered significant.

## Results

### Baseline clinical characteristics of BD patients

24 BD patients (15 men and 9 women) were all of the Han Chinese population (100%). Their mean age was, on average, 35.83 ± 11.96 years old. The median disease duration of BD was 120 months (range 13–379 months). The median age at diagnosis of BD was 28.5 years (range 17–60 years). All cases initially presented with oral ulcers. The interval between the onset of an oral ulcer and the BD diagnosis ranged from 1 to 379 months, with a median time of 71 months. The baseline clinical characteristics and medications of the BD patients are shown in Table 1. Twelve cases had been reviewed with a mean follow-up time of 7.67 ± 2.06 months. After being treated with glucocorticoids or immunosuppressants, 11 patients (91.7%) improved as measured by decreased ESR and CRP levels.

**Table 1:** Demographics and clinical characteristics of Behçet’s disease (BD) patients

Parameter	Value
Age (years), mean ± SD	35.83 ± 11.96
Age at BD diagnosis (years), median (range)	28.5 (17–60)
Disease duration (months), median (range)	120(13–379)
BDCAF, median (range)	10 (3–33)
Clinical features of BD	
Oral ulcer	24 (100%)
Genital ulcer	17 (70.8%)
Skin lesions	16 (66.7%)
Ocular lesions	8 (33.3%)
Vascular involvement	7 (29.2%)
Pathergy reaction	5 (20.8%)
Gastrointestinal involvement	3 (12.5%)
Neurologic involvement	1 (4.2%)
Erythrocyte sedimentation rate (mm/h), median (range)	15.5 (2–76)
C-reactive protein (mg/L), median (range)	9.62 (0.56–53.1)
Treatment-naive	7 (29.2%)
Current medications	
Glucocorticoid	15 (62.5%)
Thalidomide	13 (54.2%)
Cyclophosphamide	11 (45.8%)
Salazosulfapyridine	3 (12.5%)
Cyclosporine A	2 (8.3%)
Methotrexate	2(8.3%)
Azathioprine	1 (4.2%)
Etanercept	1 (4.2%)

Values are shown as *n* (%) unless otherwise indicated

*BDCAF* Behçet’s Disease Current Activity Form

### Serum metabolomics

**Fig. 1:**
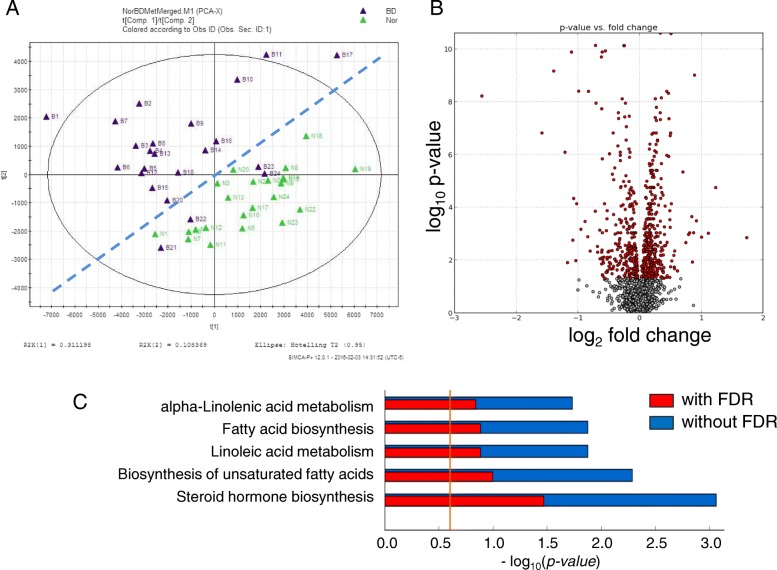
Multivariate data analyses of serum metabolomic profiles segregate Behçet’s disease (BD) patients from the healthy cohort (HC; Nor). **a** Principle components analysis (PCA) unsupervised clustering plots for healthy (green triangle) and BD (purple triangle) are shown. **b** The volcano plot displays *t* test results of samples from BD and HC. Ions marked in red show a significant difference in intensity between BD and HC. **c** KEGG pathway enrichment analysis results of the differential ions identified in the multivariate analysis of serum metabolomic profiles from HC and BD patients. FDR false discovery rate

To characterize metabolomic alterations associated with BD, we analyzed the metabolomics of BD patients and HC using a UPLC-QTOF-MS approach. Unsupervised PCA plots were generated by SIMCA-P software, and differential analysis was carried out using Metabolyzer. The volcano plot showing the differential ions between HC and BD patients is shown in Fig. 1. Individuals and variables with similar profiles are grouped together in the plot. In this PCA plot, the purple and green spots, which represent individual pretreated BD patients and HC, respectively, form two segregated clusters. Statistical analysis of the metabolomics data from pretreated BD patients and the control group revealed differential ions between these two groups with statistical significance as shown in the volcano plot (Fig. 1b). In the volcano plot, the red dots represent ions that show significantly different (*p* value less than 0.05) levels between BD patients and HC. Putative molecules of these differential ions were designated by screening the accurate mass in metabolite databases, as stated in the Methods.

KEGG pathway analysis results indicate the metabolic pathways associated with the differential metabolites. Prominent pathways with a false discovery rate (FDR)-corrected *p* value less than 0.25 are shown in Fig. 1c.

### Serum lipidomics

**Fig. 2:**
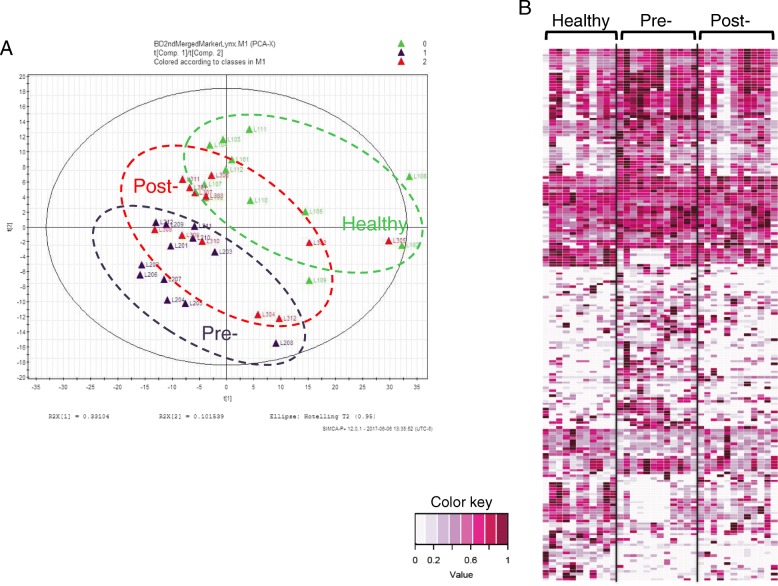
Serum lipidomic profile phenotype of treated Behçet’s disease (BD) patients moves towards that of the healthy cohort (HC). **a** PCA clustering plot of samples from these three groups with differential metabolites identified between pretreatment BD patients and HC. **b** Heat map of the metabolites with significantly different abundance between BD and HC

Since the major pathways in Fig. 1c pointed to lipid metabolism, we performed the lipidomic profiling analysis by UPLC-QTOF-MS^E^. To address whether the treatments have an effect on the disease-associated metabolomics, serum lipidomic profiling was performed using samples from twelve diagnosed patients before and after treatment, as well as a healthy cohort of the same number. Statistically significant differential metabolites between HC and pretreatment BD patients were determined using Metabolyzer. PCA analysis results for the healthy, pretreatment, and post-treatment groups based on these differential metabolites are shown in Fig. 2a. This PCA plot shows that the post-treatment cluster (red circle with a dotted line) is located in the middle, between the pretreatment cluster (dark blue circle with a dotted line) and the healthy cluster (green circle with a dotted line), suggesting a drift of diseased data points towards the direction of the healthy group after treatment. RF algorithms were used to generate a heat map of these differential metabolites for these three groups (Fig. 2b).

**Fig. 3:**
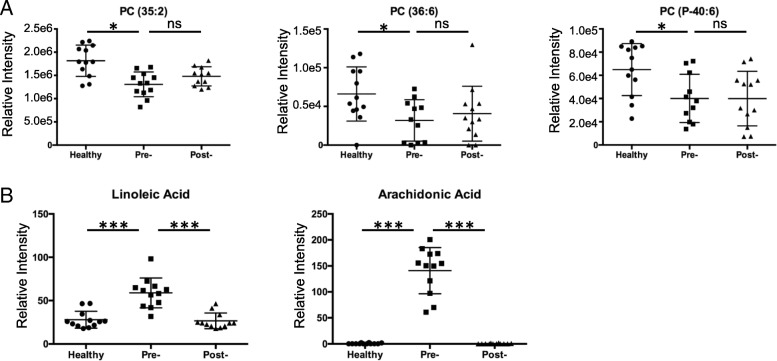
Levels of phosphatidylcholines (PCs) and polyunsaturated fatty acids (PUFAs) are affected by treatments in a different way. Comparison of the level of three PCs (**a**) and two PUFAs (**b**) in healthy controls (HC), pretreatment Behçet’s disease (BD) (Pre-) patients, and post-treatment BD (Post-) patients. The significance of metabolite levels was determined using a two-tailed student t test. *p < 0.05, ***p < 0.001. ns not significant

From the list of the differential ions, lower levels of several ions with the putative identification of phosphatidylcholine (PC) were found in the BD patient group compared with the HC group. Verification of the three selected ions was performed through MS/MS, which confirmed the identity of these ions as PCs (Additional file 1). The scatter plots in Fig. 3a show the lower level of the three PCs detected in the serum of the pretreatment BD patients compared with those in the serum of HC, but no significant difference was seen between the pretreatment and post-treatment groups.

In addition to the above phospholipids, levels of several free fatty acids from pretreatment BD patients were found to be significantly different from those of HC. We observed markedly lower levels of several polyunsaturated fatty acids (PUFAs) in the HC group compared with the pretreatment BD group, including two omega-6 (n-6) fatty acids (Fig. 3b) LA (18:2n-6) and AA (20:4n-6), and oleic acid (OA), an n-9 PUFA (Additional file 2). In contrast to the small difference between pretreatment and post-treatment BD groups for PCs, a significantly lower level of PUFAs was found in the post-treatment BD group compared with the pretreatment BD group, indicating that the treatment effectively corrected the abnormal increases of these PUFAs in BD patients. Validations of these PUFAs by MS/MS are shown in Additional file 2 and Additional file 3.

Receiver operating characteristic (ROC) analysis along with sensitivities and specificities of the area under the curve (AUC) > 0.85, is shown in Table 2 and in Additional file 4. ROC curves showed that AA was the most efficient diagnostic performance (AUC = 0.9495), compared with PCs and LA (Table 2 and Additional file 4). The sensitivity of PC(34:3), PC(40:8), LA, and AA in diagnosis of BD were comparable (0.96%, 0.88%, 0.9474%, and 0.9474%, respectively), but the specificity of PC(40:8) and AA were higher than PC(34:3) and LA (Table 2).

**Table 2:** The diagnostic values of phosphatidylcholines (PCs), arachidonic acid (AA), and linoleic acid (LA) in Behçet’s disease (BD)

	Sensitivity (%, 95% CI)	Specificity (%, 95% CI)	AUC (95% CI)	Likelihood ratio
PC(34:3)	96 (79.65–99.9)	62.5 (40.59–81.2)	0.86 (0.7595–0.9605)	2.56
PC(40:8)	88 (68.78–97.45)	83.33 (62.62–95.26)	0.8617 (0.7434–0.9800)	5.28
LA	94.74 (73.97–99.87)	64 (42.52–82.03)	0.8505 (0.7382–0.9628)	2.63
AA	94.74 (73.97–99.87)	88 (68.78–97.45)	0.9495 (0.8793–1.020)	7.89

*AUC* area under the curve, *CI* confidence interval

**Fig. 4:**
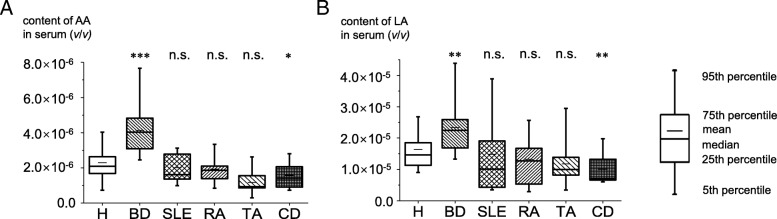
Serum level of arachidonic acid (AA) and linoleic acid (LA) in the validation cohort. Levels of AA (**a**) and LA (**b**) in patients with Behçet’s disease (BD) (*n* = 25), systemic lupus erythematosus (SLE) (*n* = 12), rheumatoid arthritis (RA) (*n* = 12), Takayasu’s arteritis (TA) (*n* = 15), and Crohn’s disease (CD) (*n* = 15), and healthy controls (HC) (*n* = 19). Statistical significance between disease groups and HC was determined using a two-tailed student *t* test. *p < 0.05, **p < 0.01, ***p < 0.001. n.s. not significant

To verify these findings, the concentrations of LA and AA were further determined using reference standards in an independent cohort containing BD, RA, SLE, TA, and CD patients and HC. As shown in Fig. 4 and Additional file 5, the serum levels of LA and AA in BD patients were significantly higher than those in HC (*p* = 2.35 × 10^−3^ and *p* = 6.1 × 10^−6^). ROC curves were also made to indicate their diagnostic efficiency (Table 2 and Additional file 4). The serum levels of LA and AA in patients with RA, SLE, and TA were comparable with those in HC (Fig. 4). In addition, the levels of LA and AA in BD patients were significantly higher than they were in disease controls, suggesting that LA and AA might serve as specific markers for BD. Intriguingly, the serum levels of LA and AA in patients with CD were significantly lower than those in HC (*p* = 0.016 and *p* = 0.002, respectively) which need further investigation.

## Discussion

BD is a chronic autoimmune disease characterized by various clinical manifestations that may be similar to other diseases. Given the lack of specific serological markers, it is difficult to diagnose the disease early and to treat it. This study is the first to use a metabolomics approach for exploring the potential diagnostic markers of BD. Our study suggests that the altered levels of PCs and PUFAs may be indicative of the diagnosis of BD. Two n-6 fatty acids, LA and AA, may provide insight into therapeutic effects.

PCs, the major structural components of cell membranes, serve as fatty acid carriers and play an important role in metabolism and signaling [18, 19]. Table 3 lists PC and lysophosphatidylcholine (LPC) species that showed significantly different serum levels in BD patients compared with HC. PCs have been studied as potential metabolic biomarkers for the diagnosis of several diseases, such as calcific coronary artery disease [20] and endometriosis [21]. Since PCs are involved in pathogenic processes such as chronic inflammation, autoimmunity, and allergy [22–24], it has been suggested that PCs act as predictive metabolites corresponding with the activation of inflammatory, oxidant, and fibrotic pathways in progressive nephropathy [22]. Increased levels of various polyunsaturated PCs were positively associated with asthma [23]. In addition, PC/LPC ratios in plasma may be indicators of the early stages of RA, and they may be a reliable measure of inflammation. PC/LPC ratios could increase on therapy with tumor necrosis factor (TNF)α inhibitors [24]. In our study, the levels of PCs, PC(35:2), PC(36:6), and PC(P-40:6) in the pretreated BD group were lower than in the HC group. This may be associated with the hyperinflammatory status of BD. Intriguingly, however, the decreased level of PCs did not recover after glucocorticoid or immunosuppressant treatment. The implications of PCs in the pathophysiology of BD need to be further studied.

**Table 3:** The levels of ions with putative phosphatidylcholine (PC) identification in Behçet’s disease (BD) patients and healthy controls (HC)

Ion (mass_retention time)	PC (putative)	Student’s *t* test	Welch’s *t* test	Log2 fold change (BD/normal)
734.5692_5.3326	PC(32:0)	0.0325	0.0321	0.145
732.5534_4.9313	PC(32:1)	0.0090	0.0082	0.327
756.5523_4.6252	PC(34:3)	0.0001	0.0001	−0.869
754.5359_4.4534	PC(34:4)	0.0111	0.0119	−0.319
772.5835_5.2313	PC(35:2)	0.0463	0.0356	−0.641
768.5513_4.7218	PC(35:4)	0.0289	0.0264	−0.265
778.5369_4.3239	PC(36:6)	0.0170	0.0169	−0.825
810.5997_5.4325	PC(38:4)	0.0039	0.0038	−0.175
806.5683_4.8573	PC(38:6)	0.0000	0.0000	−0.366
804.5523_4.4254	PC(38:7)	0.0000	0.0000	−0.662
820.5828_5.0949	PC(39:6)	0.0008	0.0004	−0.472
834.5997_5.318	PC(40:6)	0.0023	0.0025	−0.299
832.5836_4.8989	PC(40:7)	0.0001	0.0001	−0.433
832.5815_5.43	PC(40:7)	0.0357	0.0340	−0.164
830.563_4.5397	PC(40:8)	0.0000	0.0000	−0.418
828.5502_4.8787	PC(40:9)	0.0003	0.0003	−0.325
856.5814_5.3104	PC(42:9)	0.0002	0.0002	−0.481
904.5899_4.9432	PC(44:10)	0.0188	0.0197	−0.210
796.6189_5.5595	PC(O-38:4)	0.0223	0.0213	0.191
792.5885_5.1251	PC(O-38:6)	0.0066	0.0068	−0.578
790.5699_5.0568	PC(P-38:6)	0.0122	0.0114	−0.265
818.6028_5.1752	PC(P-40:6)	0.0439	0.0451	−0.208
524.371_1.7951	LysoPC(18:0)	0.0048	0.0050	−0.264
524.3711_1.9491	LysoPC(18:0)	0.0256	0.0185	−0.182
518.3219_1.3367	LysoPC(18:3)	0.0186	0.0169	−0.390
568.34_1.0332	LysoPC(22:6)	0.0001	0.0001	−0.430

The *p* values of the Student *t* test and Welch’s *t* test are shown

Ions with a *p* value of less than 0.05 are included

The n-6 and n-3 PUFAs play an important role in the regulation of biological functions, inflammation, and immunity. Eicosanoids derived from n-6 PUFAs have a proinflammatory role, while those derived from n-3 PUFAs have an anti-inflammatory role [25]. It has been suggested that inflammatory and autoimmune diseases can be managed by regulating the intake of n-3 and n-6 PUFAs in the diet. In fact, modulation of the n-6/n-3 PUFA proportion is beneficial in several diseases, such as RA, ulcerative colitis, and cardiovascular diseases. It can decrease disease activity and minimize the requirements for anti-inflammatory drugs [26–29].

LA, one of the n-6 PUFAs, is an essential fatty acid because it cannot be synthesized in the human body. LA can be converted to the metabolically active AA, an n-6 PUFA that is present in the phospholipids of biomembranes. AA can be metabolized to several proinflammatory eicosanoids via multiple metabolic pathways, including the cyclooxygenase, lipoxygenase, and cytochrome P450 monooxygenases pathways [30]. AA can be involved in the regulation of inflammation through its eicosanoid metabolites, such as prostaglandin E2, thromboxane A2, and leukotriene B4 [31]. It is reported that AA-derived eicosanoids can reduce inflammatory Th17 and Th1 cell-mediated inflammation and improve colitis-associated immunopathology [32]. In our study, increased levels of LA and AA were found in pretreated BD patients compared with HC. This may reflect enhanced inflammation and relate to the occurrence and development of the disease. Our results showed a reduced level of two n-6 PUFAs in post-treatment BD patients, which indicated that these PUFAs, as indicators of inflammatory symptoms, may be useful for treatment assessment.

OA, an n-9 PUFA, is present in human plasma, cell membranes, and adipose tissue. OA can regulate physiological and pathological changes in cells through cell surface receptors or nuclear receptors [33, 34]. OA has been linked with metabolic and inflammatory diseases, and OA induces neutrophil accumulation and the release of inflammatory cytokines [35]. OA can also sensitize dendritic cells, resulting in augmented secretion of Th1/17 cytokines upon proinflammatory stimulation, and it can further promote an inflammatory response [36]. Our study suggests that OA may provide insights for the diagnosis and therapeutic effects of BD.

Ahn et al. [37] recently reported that the serum metabolite profiles of BD patients were distinctively separate from those of HC using gas chromatography with time-of-flight mass spectrometry (GC/TOF-MS). Five metabolic biomarkers, namely decanoic acid, fructose, tagatose, LA, and OA, were selected and validated as potential metabolite biomarkers for diagnosing BD. While GC/MS and LC/MS can be complimentary in terms of detecting different metabolites, the application of GC is limited to those who are volatile before or after derivatization. Our metabolomics profiling pointed to a different lipid metabolism in BD patients, so we designed UPLC-QTOF-MS^E^ methods for lipidomics, which was not included in the study of Ahn et al. These differences in analytical methods may lead to different biomarkers from the previous study [37]. In addition to identifying the differential metabolites between BD patients and HC, we have also compared lipidomic profiles before and after treatment to search for potential biomarkers with therapeutic effects.

To further assess the diagnostic efficiency of these biomarkers, an independent validation cohort was employed. Since all patients were enrolled from a single center with relatively small sample sizes, we cannot exclude the possibility that our conclusions may have some specific limitations to the Chinese population. A multicenter study with a large sample size would therefore strengthen this study. In addition, we found that serum levels of LA and AA could distinguish BD patients from HC and other inflammatory or autoimmune diseases, including RA, SLE, TA, and CD, suggesting that these serum biomarkers might be specific markers for BD diagnosis.

## Conclusions

In conclusion, our study supports the importance of PCs, LA, AA, and OA in the diagnosis and therapeutic effects of BD. This study is the first to use a metabolomic approach in the study of BD. Further investigations are required to explore the implication of these metabolomics alterations in the pathophysiology of BD.

## Additional files

Additional file 1: Verification of PCs by multiple reaction monitoring. These panels show MS/MS spectra of the indicated ions. Multiple reaction monitoring transitions were monitored for PC signature fragmentation (m/z 184). (PDF 46 kb)

Additional file 2: Verification of PUFAs by MS/MS. Retention time of two n-6 PUFAs, linoleic acid and arachidonic acid were compared with that of the pure chemicals. MS/MS spectra are shown. (PDF 46 kb)

Additional file 3: Treatment reverses the increased level of oleic acid in serum. (A) Abundance of oleic acid in healthy volunteers, pretreatment BD (Pre-) patients, and post-treatment BD (Post-) patients. ***p < 0.001. (B) Verification of oleic acid by MS/MS. (PDF 59 kb)

Additional file 4: The ROC curve of PCs, AA, and LA in BD patients. (A) The ROC curve of PCs with area under the curve (AUC) > 0.85 in BD patients. (B) The ROC curve of AA and LA in BD patients. (PDF 18 kb)

Additional file 5: Comparison of the content of AA and LA in an independent cohort. (A) Identification of AA and LA in serum samples by comparison with reference standards. (B) Calibration curves of AA and LA with rosmarinic acid as the internal standard. (C) Contents of LA and AA in BD and HC samples. (D) Correlation of ESR and CRP with the serum levels of LA and AA in BD. (PDF 253 kb)

## Abbreviations

AA: Arachidonic acid
AUC: Area under the curve
BD: Behçet’s disease
BDCAF 2006: BD Current Activity Form 2006
CD: Crohn’s disease
CRP: C-reactive protein
ESR: Erythrocyte sedimentation rate
GC: Gas chromatography
HC: Healthy controls
ICBD: International Criteria for Behçet’s Disease
IS: Internal standard
KEGG: Kyoto Encyclopaedia of Genes and Genomes
LA: Linoleic acid
LC: Liquid chromatography
LPC: Lysophosphatidylcholine
MS/MS: Tandem mass spectrometry
OA: Oleic acid
PC: Phosphatidylcholine
PCA: Principal component analysis
PUFA: Polyunsaturated fatty acid
PUMCH: Peking Union Medical College Hospital
RA: Rheumatoid arthritis
RF: Random Forests
ROC: Receiver operating characteristic
SLE: Systemic lupus erythematosus
TA: Takayasu’s arteritis
UPLC-QTOF-MS: Ultra-performance liquid chromatography-quadrupole time-of-flight mass spectrometry
