# Supplementary figures and images for: Metabolomic alterations associated with Behçet’s disease
Source: Arthritis Res Ther. 2018 Sep 24;20:214. doi: 10.1186/s13075-018-1712-y (PMC6154820; doi:10.1186/s13075-018-1712-y)

### PC (35:2)

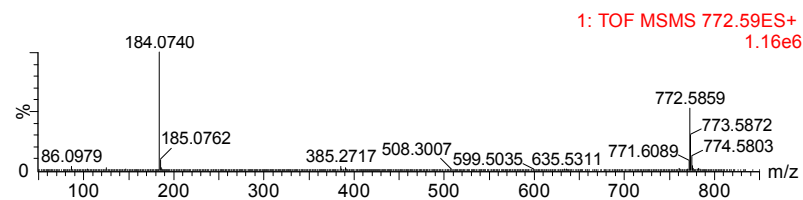

### PC (36:6)

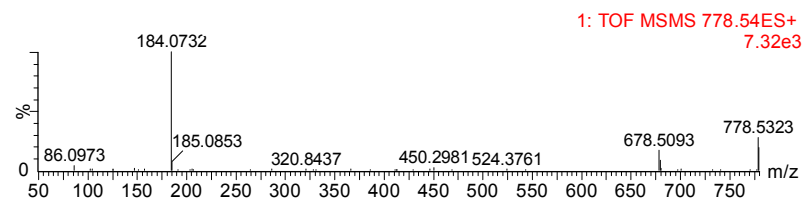

### PC (P-40:6)

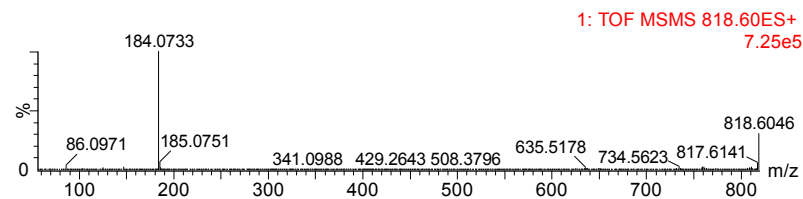

Additional file 1

Supplement: Supplementary file 1 — Verification of PCs by multiple reaction monitoring. These panels show MS/MS spectra of the indicated ions. Multiple reaction monitoring transitions were monitored for PC signature fragmentation (m/z 184). (PDF 46 kb) [file 13075_2018_1712_MOESM1_ESM.pdf]

# Linoleic Acid

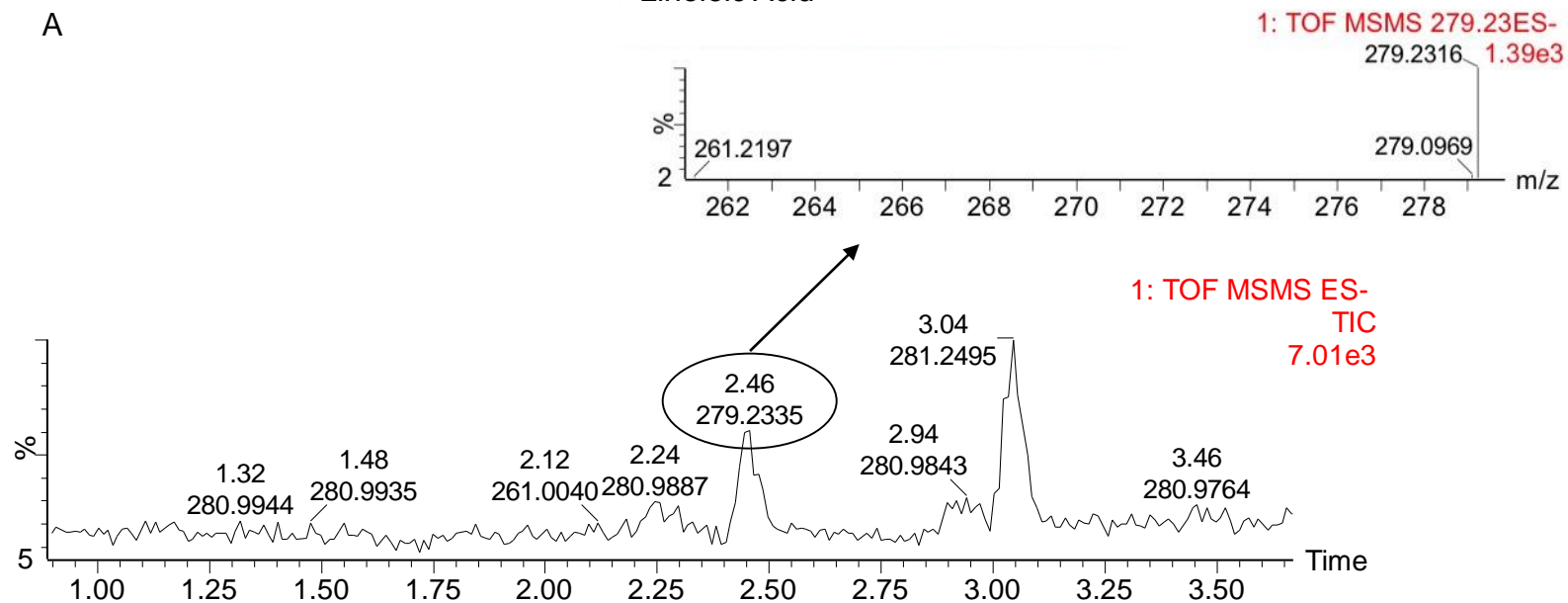

# Arachidonic Acid

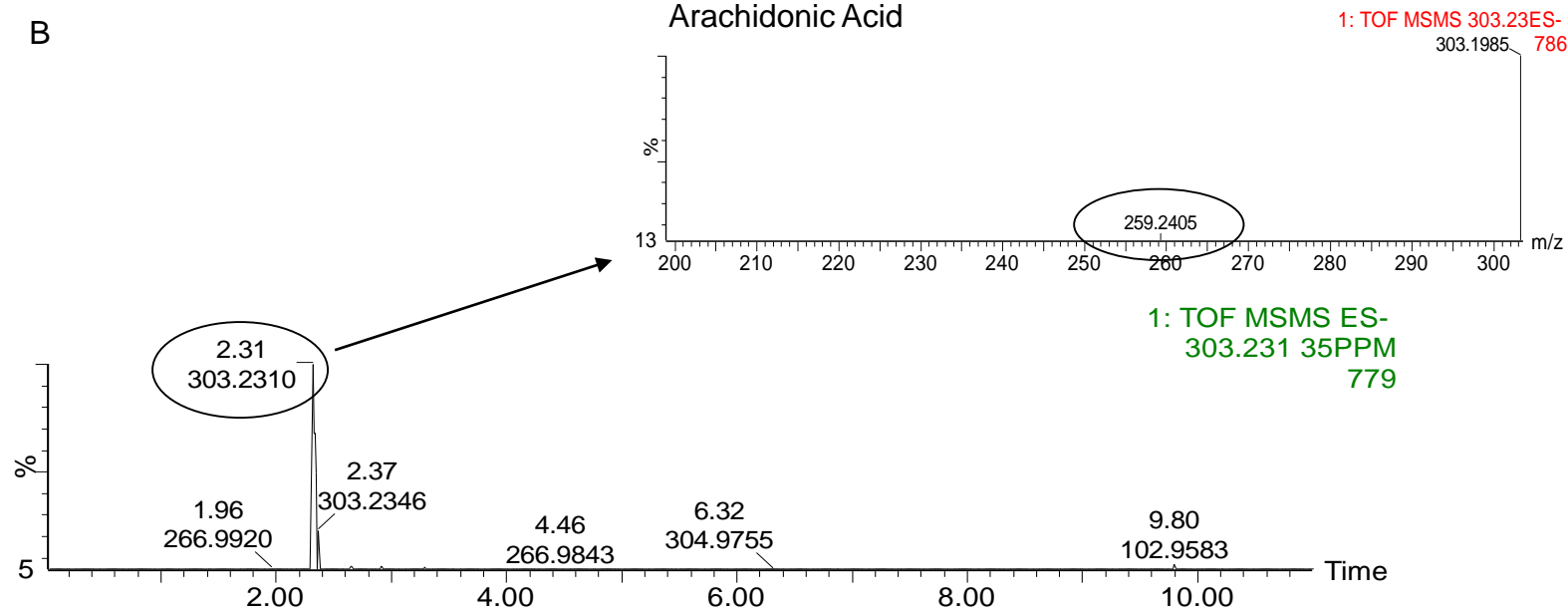

Supplement: Supplementary file 2 — Verification of PUFAs by MS/MS. Retention time of two n-6 PUFAs, linoleic acid and arachidonic acid were compared with that of the pure chemicals. MS/MS spectra are shown. (PDF 46 kb) [file 13075_2018_1712_MOESM2_ESM.pdf]

A

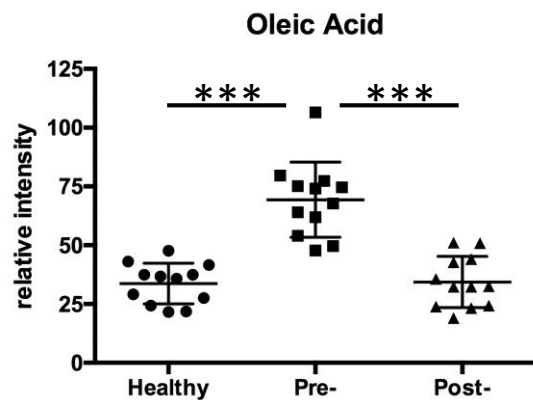

B

Oleic Acid

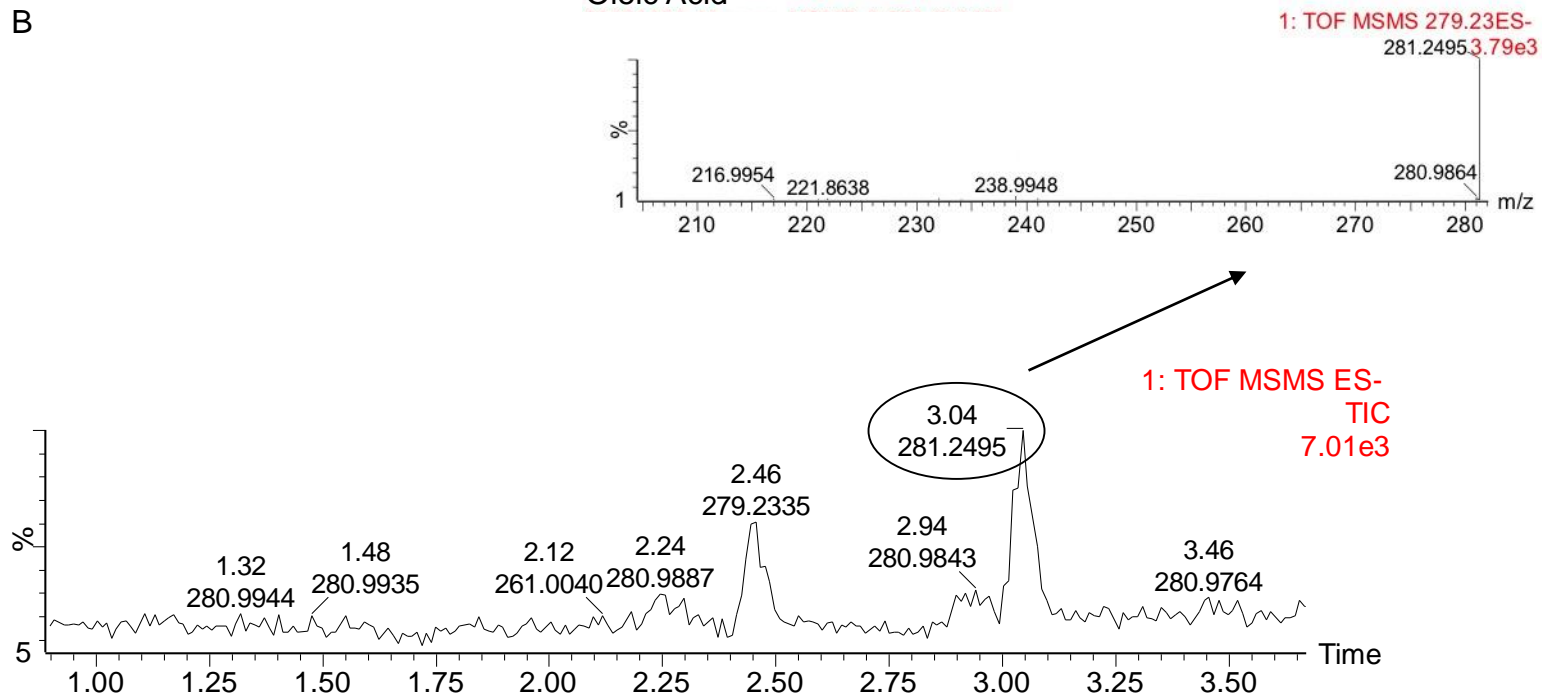

Supplement: Supplementary file 3 — Treatment reverses the increased level of oleic acid in serum. (A) Abundance of oleic acid in healthy volunteers, pretreatment BD (Pre-) patients, and post-treatment BD (Post-) patients. ***p < 0.001. (B) Verification of oleic acid by MS/MS. (PDF 59 kb) [file 13075_2018_1712_MOESM3_ESM.pdf]

A

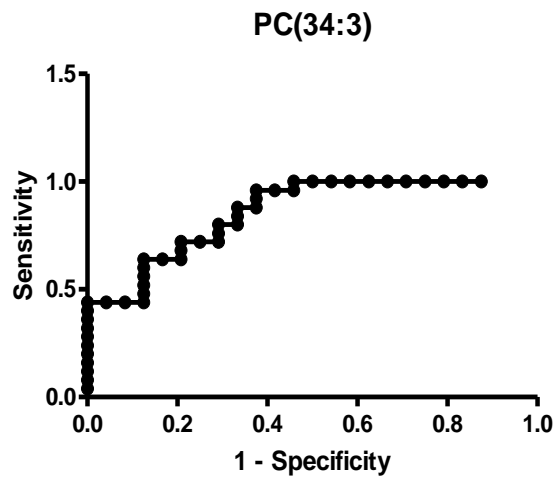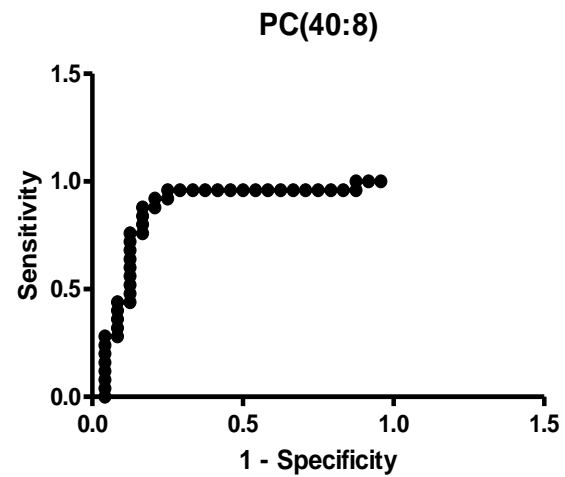

B

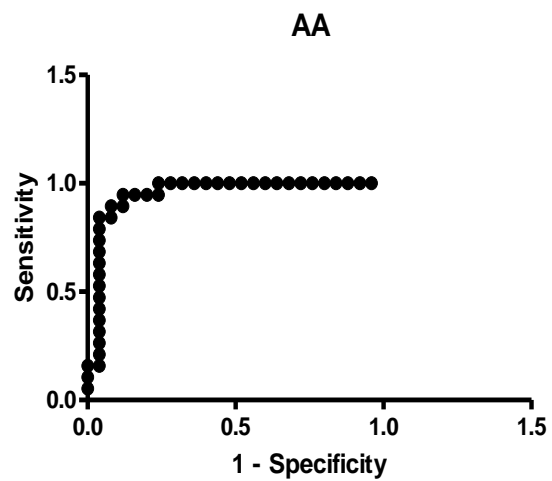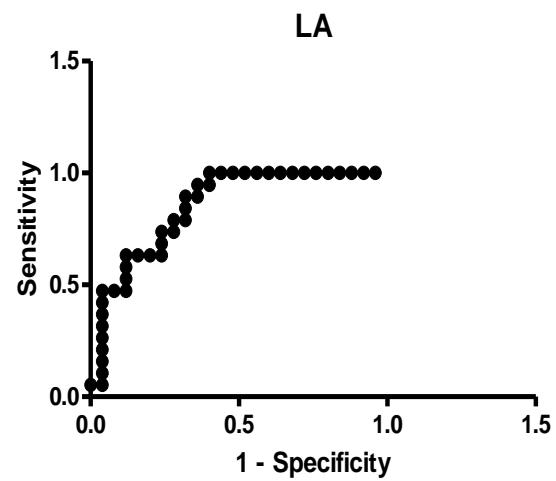

Supplement: Supplementary file 4 — The ROC curve of PCs, AA, and LA in BD patients. (A) The ROC curve of PCs with area under the curve (AUC) > 0.85 in BD patients. (B) The ROC curve of AA and LA in BD patients. (PDF 18 kb) [file 13075_2018_1712_MOESM4_ESM.pdf]
