# Supplementary material for: Metabolomic alterations associated with Behçet’s disease
Source: Arthritis Res Ther. 2018 Sep 24;20:214. doi: 10.1186/s13075-018-1712-y (PMC6154820; doi:10.1186/s13075-018-1712-y)
Supplement: Supplementary file 5 — Comparison of the content of AA and LA in an independent cohort. (A) Identification of AA and LA in serum samples by comparison with reference standards. (B) Calibration curves of AA and LA with rosmarinic acid as the internal standard. (C) Contents of LA and AA in BD and HC samples. (D) Correlation of ESR and CRP with the serum levels of LA and AA in BD. (PDF 253 kb) [file 13075_2018_1712_MOESM5_ESM.pdf]

A

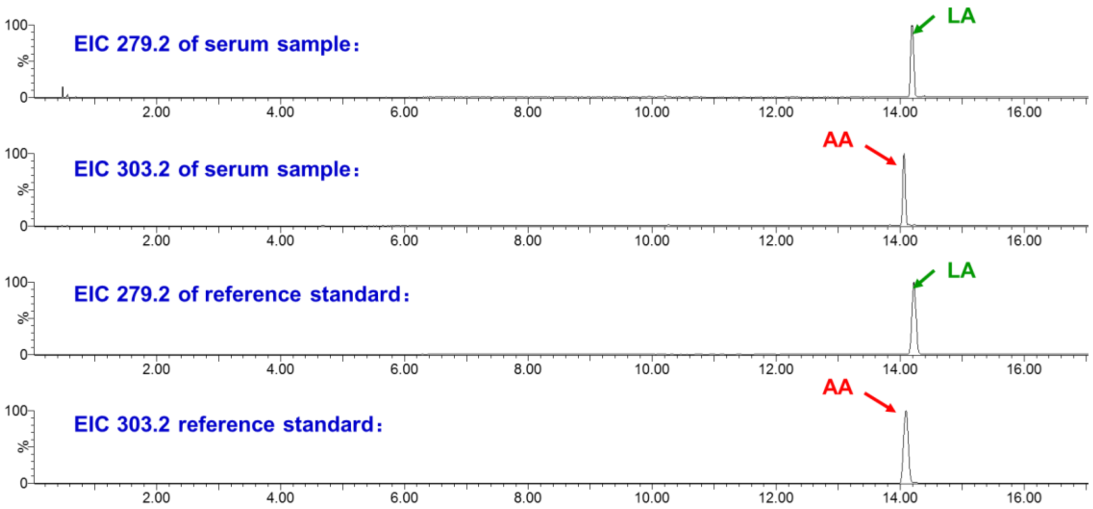

B

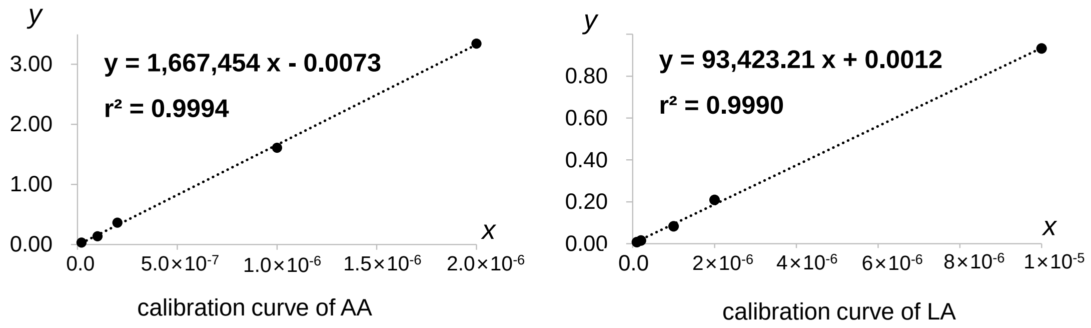

C

Contents of LA and AA in BD and HC samples

sample ID	sex*	age	hCRP	ESR	BDCAF 2006	IgG	IgA	IgM	content of AA (v/v)	content of LA (v/v)
BD-1	M	19	64.91	53	4	10.76	3.1	1.22	0.00048%	0.00222%
BD-2	F	16	15.92	36	2	19.37	4.1	1.1	0.00050%	0.00238%
BD-3	F	56	6.68	26	4	17.04	3.72	0.61	0.00049%	0.00157%
BD-4	M	45	18.23	18	1				0.00044%	0.00259%
BD-5	M	33	26.77	81	2	27.04	3.01	1.51	0.00051%	0.00278%
BD-6	M	25	133.34	32	3	14.39	3.48	1.1	0.00030%	0.00157%
BD-7	M	31	15.88	15		16.26	4.71	0.83	0.00040%	0.00224%
BD-8	M	42	18.99	20	2	11.29	3.42	1.37	0.00027%	0.00169%
BD-9	F	43	7.2	44	1	14.73	3.35	1.63	0.00035%	0.00154%
BD-10	M	30	13.47	53	1	18.94	1.64	1.41	0.00048%	0.00184%
BD-11	F	23	10.09	25	2	14.26	4.56	1.52	0.00038%	0.00133%
BD-12	F	32	8	7	2	10.7	2.06	1.32	0.00038%	0.00254%
BD-13	M	38	0.86	10	3	15.93	5.15	1.21	0.00083%	0.00439%

BD-14	M	55	1.86	3	1				0.00025%	0.00246%
BD-15	F	19	3.73	20	2				0.00035%	0.00211%
BD-16	F	15	1.5	14	2	15.98	2.84	1.43	0.00015%	0.00084%
BD-17	F	23	0.06	2	2				0.00043%	0.00491%
BD-18	F	45	1.26	20	3	10.48	1.82	2.09	0.00026%	0.00171%
BD-19	F	40	1.18	12	2	13.14	1.78	1.54	0.00077%	0.00314%
BD-20	M	34	4.03	11	2	13.84	3.09	1.47	0.00031%	0.00209%
BD-21	F	13	0.29	5	1	12.27	2.29	2.58	0.00038%	0.00136%
BD-22	M	24	7.22	6	4	10.5	2.23	0.46	0.00043%	0.00257%
BD-23	M	67	1.44	14	4	13.87	2.15	2.09	0.00040%	0.00229%
BD-24	F	46	2.03	15	4	11.35	5.06	1.15	0.00029%	0.00291%
BD-25	F	46	1.22	8	1	12.61	3.71	0.51	0.00051%	0.00332%
HC-1									0.00020%	0.00127%
HC-2									0.00020%	0.00111%
HC-3									0.00021%	0.00178%
HC-4									0.00022%	0.00128%
HC-5									0.00026%	0.00108%
HC-6									0.00007%	0.00142%
HC-7									0.00016%	0.00091%
HC-8									0.00024%	0.00121%
HC-9									0.00017%	0.00210%
HC-10									0.00025%	0.00113%
HC-11									0.00016%	0.00108%
HC-12									0.00021%	0.00169%
HC-13									0.00021%	0.00142%
HC-14									0.00024%	0.00165%
HC-15									0.00017%	0.00146%
HC-16									0.00013%	0.00162%
HC-17									0.00018%	0.00182%
HC-18									0.00030%	0.00185%
HC-19									0.00006%	0.00084%

* Note: M, male; F, female.

D

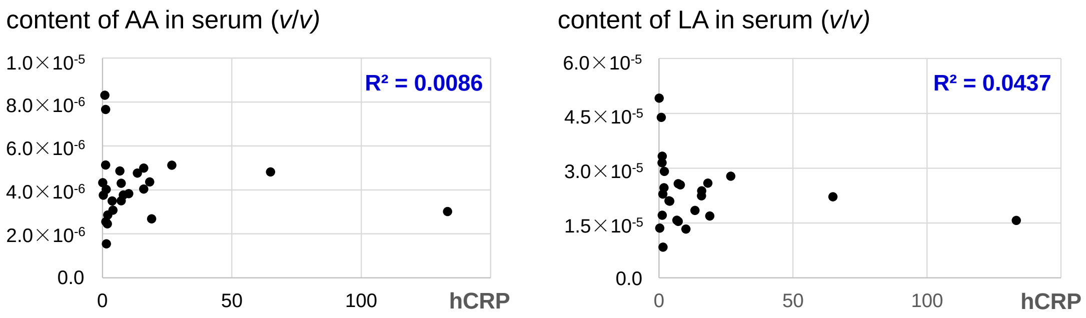

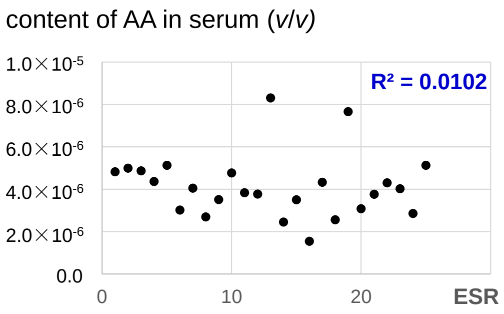
content of AA in serum (v/v)
1.0×10-5
8.0×10-6
6.0×10-6
4.0×10-6
2.0×10-6
0.0
0
10
20
ESR
R² = 0.0102

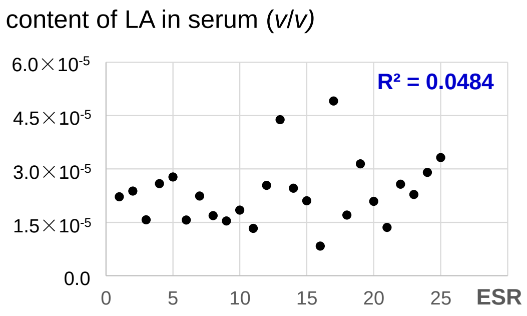
content of LA in serum (v/v)
6.0×10-5
4.5×10-5
3.0×10-5
1.5×10-5
0.0
0
5
10
15
20
25
ESR
R² = 0.0484
